# Computerized analysis of snoring in Sleep Apnea Syndrome

**DOI:** 10.1590/S1808-86942011000400013

**Published:** 2015-10-19

**Authors:** Fabio Koiti Shiomi, Ivan Torres Pisa, Carlos José Reis de Campos

**Affiliations:** 1 MSc, professor at the Instituto Federal de Educação Tecnológica de São Paulo (IFSP); 2 Doctoral degree, adjunct professor in the graduate program of the Health Informatics Department, UNIFESP; 3 Doctoral degree, tutoring professor in the graduate program of the Health Informatics Department, UNIFESP

**Keywords:** apnea, decision support techniques, information systems, sleep apnea syndromes, snoring

## Abstract

**Abstract:**

The International Classification of Sleep Disorders lists 90 disorders. Manifestations, such as snoring, are important signs in the diagnosis of the Obstructive Sleep Apnea Syndrome; they are also socially undesirable.

**Objective:**

The aim of this paper was to present and evaluate a computerized tool that automatically identifies snoring and highlights the importance of establishing the duration of each snoring event in OSA patients.

**Material and Methods:**

The low-sampling (200 Hz) electrical signal that indicates snoring was measured during polysomnography. The snoring sound of 31 patients was automatically classified by the software. The Kappa approach was applied to measure agreement between the automatic detection software and a trained observer. Student's T test was applied to evaluate differences in the duration of snoring episodes among simple snorers and OSA snorers.

**Results:**

Of a total 43,976 snoring episodes, the software sensitivity was 99.26%, the specificity was 97.35%, and Kappa was 0.96. We found a statistically significant difference (*p* <0.0001) in the duration of snoring episodes (simple snoring x OSA snorers).

**Conclusion:**

This computer software makes it easier to generate quantitative reports of snoring, thereby reducing manual labor.

## INTRODUCTION

At present, the International Classification of sleep disorders lists about 90 disorders^1^, including snoring, the obstructive sleep apnea hypopnea syndrome (OSAHS), insomnia, narcolepsy, bruxism, restless leg syndrome, and others. It is important to study these disorders, as human adults sleep about 7.5 h to 8.5 h daily.

Snoring may be defined as noisy breathing produced by vibration of soft tissues in the oropharynx^2^. Systemic arterial hypertension, heart conditions, angina, and strokes are more frequent in snorers^3^. Snoring is a relevant diagnostic sign of the obstructive apnea syndrome, as most apneic patients snore^4^. Depending on the age, up to 62% of men and 45% of women snore regularly.

Snoring is a social inconvenience; the noise makes it difficult for spouses, bed partners, or roommates to sleep. Bed partners of snorers may have poor quality sleep^5^, and may develop secondary sleep disorders^6^. A snorer may become socially unacceptable, loss of harmony in marriage, divorce, aggression, and even homicide may ensue^7^. There is evidence that people chronically exposed to snorers tend to have presbycusis (age-related hearing loss)^8^.

Polysomnography is the gold standard in the diagnosis of respiratory sleep disorders. At present, technicians in sleep clinics that offer polysomnography describe snoring subjectively - patients are described as non-snorers, moderate snorers, or snorers. In a few cases, an electric recording of snores is made; in this case, technicians may manually identify and classify snoring events during polysomnography. This method is time-consuming, expensive, and inconsistent.

The treatment of snoring consists of behavioral measures, CPAP, and surgery. Monitoring of these cases is generally subjective - done by the spouse or partner.

A few researchers have measure the intensity of sound^9^, the energy of sound, and the zero passage rate^10^ to quantify snoring more objectively and automatically, and to associate it with OSAHS. However, these values depend on the microphone, the amplifier, the A/D converter, and the distance between the patient and the microphone. Furthermore, the signal energy intensity may vary with time in the same patient^10^.

A few voice recognition techniques have been applied in the detection of snores and OSAHS; these include 500 Hz sub-band energy distribution analysis^11^, the Hidden Markov Models (HMM), a statistical technique to model, classify, and segment samples of a time series^12^, and Linear Predictive Coding^13^. These algorithms require snoring sounds to be recorded at a sampling rate above 7500 Hz^4,^^14^.

Most of the polysomnography devices in Brazil do not record or count snoring events. However, these devices generally have extra channels that are not used. Although these channels do not record snoring sounds (because of their low sampling rate - usually from 256 Hz to 2,000 Hz), electrical recording of snores may be done by using those extra channels. A low-cost piezoelectric microphone in contact with the skin and connected to a low sampling rate may be used to make an electrical recording of snores. This is not done, however, because it is complex to manually identify and quantify snoring events recorded in polysomnography.

It is reasonable to develop an open source computer code for quantifying snoring based on a low sampling rate electric signal because electric recordings of snores can be made using the installed base in polysomnography laboratories - therefore without major costs or changes in routines - and especially because information about the quality of snoring is relevant for clinical practice and science.

Several researchers have correlated snoring and its intensity^15^, ^16^, ^17^, ^18^; however, few studies have investigated the importance of the duration of snoring events in patients with or with no apnea, possibly because this information is difficult to extract and quantify.

The purpose of this paper is to introduce a tool that automatically identifies snores, to underline the importance of quantifying the duration of each snoring event in OSAHS patients, and to point out other events that may be found in the signal and that may contaminate the electric snoring signal, such as voice, coughing, and other artifacts.

## MATERIAL AND METHODS

### Description of the study population

The study sample in this preliminary study comprised 31 patients that presented 43,976 snoring events. Each patient underwent a single polysomnography. Exams were carried out in Sao Paulo at night from April 2009 to May 2009. The exam was done from 10 p.m. to 6 a.m. The age of subjects ranged from 5 to 64 years (mean - 41 years). The percentage of male subjects was 74% (23/31), and the percentage of female patients was 26% (8/31). The institutional review board of UNIFESP approved this study (no. 0896/08).

### Polysomnography

Cardio-respiratory and electroencephalographic recording were made with a BrainNet BNT-POLI device (EMSA - Equipamentos Médicos, Brazil) running the Captacoes proprietary software (data acquisition) and the Poliwin software for evaluating the exams. Acquisition and evaluation of polysomnography was done based on the American Sleep Disorders Association instruction guide. Recording were made of the following channels: EEG (electroencephalogram), ECG (electrocardiogram), EOG (bilateral electrooculogram), EMG (electromyogram), belts measuring chest wall and abdominal wall movements, nasal air flow by thermistor, oxygen saturation, and the equivalent electric snoring signal.

Sleep parameters were taken from the official report; confidentiality of the information was respected.

### Recordings of snores

The original recordings of snores were made using a small (about 5 mm diameter) common microphone with a 50-16,000 Hz response frequency, which was attached to the skin of the neck with Micropore^U+000AE^ adhesive tape (3M^U+000AE^). All signals, including snoring signals, were fed into a analog/digital converter with a 200 Hz sampling rate (each channel has 200 points or samaples per second).

The digital values were saved as a file; the contents of this file were organized as a proprietary protocol (.plg) defined by EMSA, a Brazilian manufacturer of medical devices.

### Computer program (algorithm) - snore detector

**Figure 1:**
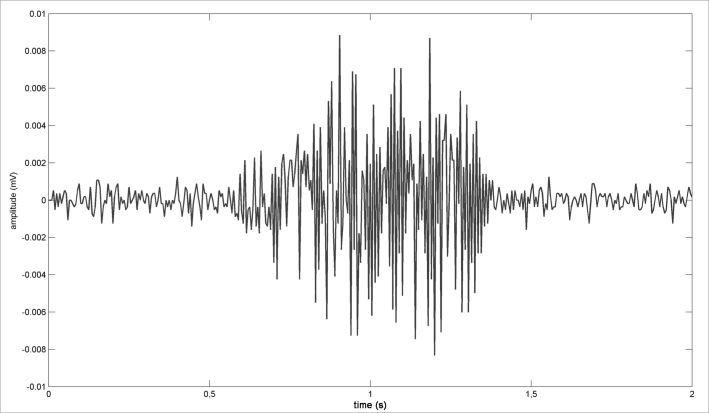
Snoring signal - Example of a snoring electric signal.

**Figure 2:**
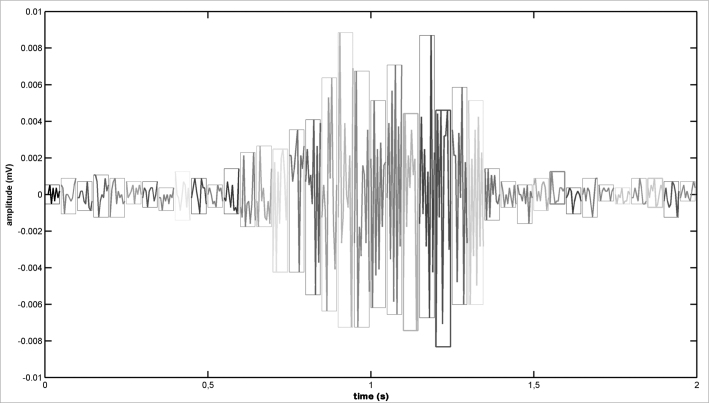
Sampled snoring signal - Sampled snoring signal (windows).
2 The effective value of each 100 ms window is computed (Figure 3);

A Hsu algorithm^19^ was used in detecting snores and quantifying the duration of each event. This algorithm consists of four steps:

1 The snoring signal is sampled in 100 ms windows (Figure 1; Figure 2);

**Figure 3:**
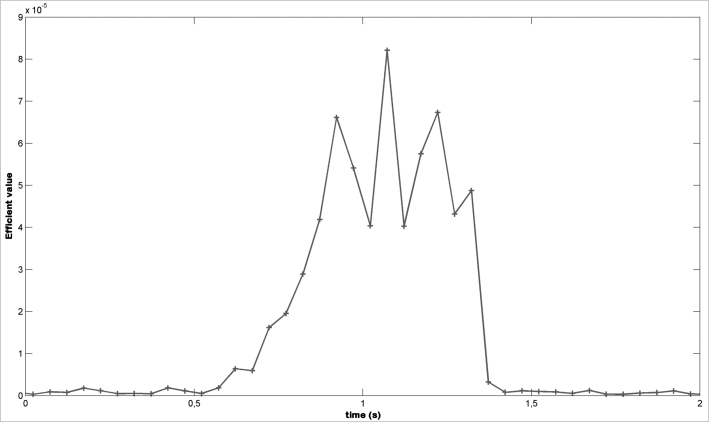
Effective value of the snoring signal - Example of calculating the effective value of the snoring signal.
3 A low-pass filter (moving-average filter) is applied using a 10-sample delay window (Figure 4);

**Figure 4:**
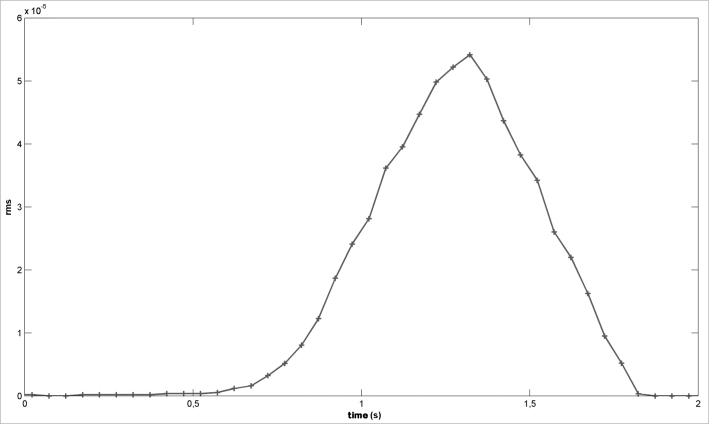
Moving-Average filter - Snoring signal after applying the moving-average filter.
4 A time series was made to define whether the electric signal generated in step 2 was compatible with snoring. This analysis identifies the beginning and the end of the snoring event. With this information (beginning and end) we calculated the duration (T_duration_) of the electric event. If the duration (T_duration_) of the event was within 0.6 to 2 seconds, it was classified as snoring.

If an electric event did not match the duration (0.6 to 2 seconds), it was marked as a probable non-snoring event. Spurious sounds, such as voice or coughing, may contaminate these recordings and generate undesired events or artifacts.

The abovementioned algorithm was implemented in MatLab. The result of the algorithmic classification was fed into the JBioSignal Viewer software.

### JBioSignal Viewer - validating the automatic snore detector

**Figure 5:**
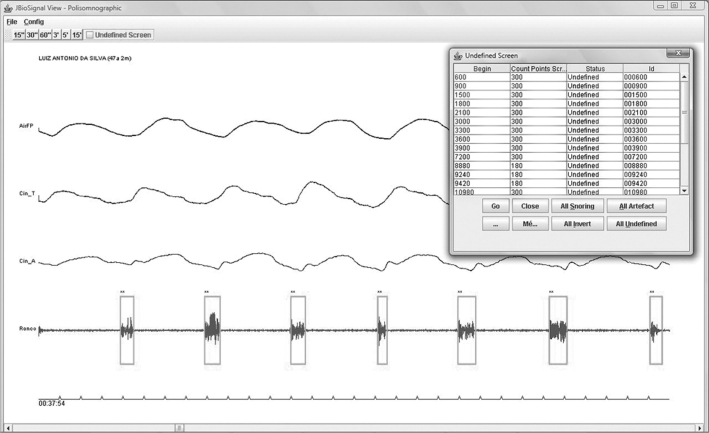
JbioSignal Viewer - Polysomnography exam viewer that was used to validate snoring episodes.

A biological signal viewer was developed in Java^U+000AE^ (Oracle) - within the Eclipse^U+000AE^ (IBM) development environment - so that trained observers could validate electric events as snoring or non-snoring). Figure 5 shows a screen of the JBioSignal Viewer.

The JBioSignal viewer marks in green (rectangle) if the algorithm classified an electric event as snoring (R); non-snoring events are marked black (NR).

All snores and artifacts detected by the algorithm were validated objectively by a trained observer, using the JBioSignal viewer, to assess the automatic snore detector.

During the evaluations, if the algorithm classified an event as a snore (green rectangle), but the trained observer considered it non-snoring, the observer was able to change the mark by clicking twice (from snore to non-snore); in this case, the rectangle became red. A mark in red indicated a difference between the algorithm (that identified a snore) and the observer (that identified a non-snore).

If the algorithm classified an event as a non-snore (black rectangle), but the trained observer considered it a snore, the observer could change the mark by clicking twice; in this case, the rectangle became purple. A mark in purple indicated a difference between the algorithm (that identified a non-snore) and the observer (that identified a snore).

**Figure 6:**
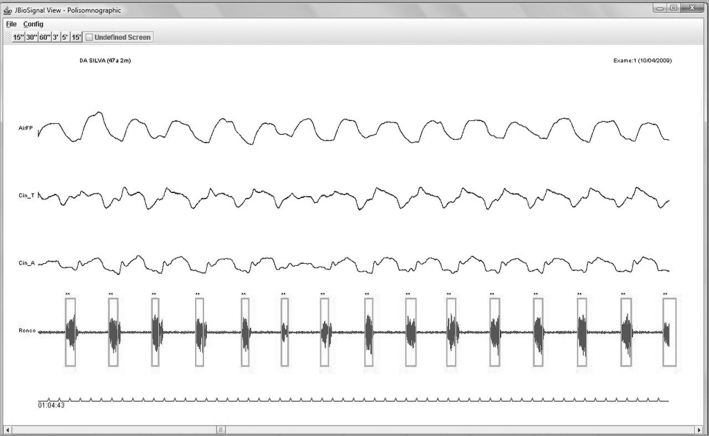
Snoring in phase with the chest and abdominal wall movements and nasal flow - Example of a snoring electric signal in phase with the chest and abdominal wall movements and nasal flow.
2) The shape of the snoring signal should fit into the pattern marked by the rectangle in green, as in Figure 6;
3) The duration of snores should be within 0.6 to 2 seconds;
4) The signal amplitude should be at least double that of silence. Figure 6 shows this ratio;
5) Patients should be sleeping, a condition which was assured by taking into account the hypnogram of the medical report when making a visual judgment.

To reduce uncertainty when making a visual judgment on the automatic detector, trained observers applied the following inclusion criteria to validate a given electric event as a snore:

1) The electric event (snore) should be in phase with the nasal airflow signal, and the signals from the belts measuring chest wall and abdominal wall movements. Figure 6 shows snoring events in phase with nasal airflow, and chest wall and abdominal wall movements;

Trained observers validated each snore (S) that was classified as such by the automatic snore detector. If any of these four criteria were not met, the event was considered a non-snore (NS).

**Figure 7:**
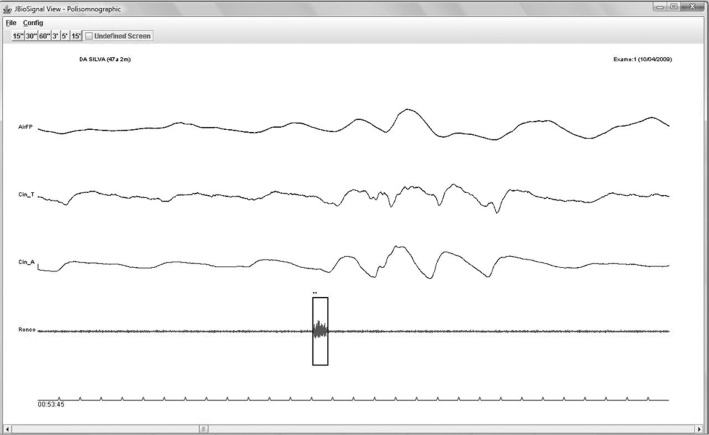
Artifact (non-snore) - Event classified as an artifact.

An isolated electric event that fit into the five criteria was defined as a non-snore (NS), as shown in Figure 7.

### Statistics

Results were expressed as percentages, means, and standard deviation. We used variables with a normal (Gaussian) distribution when applying the parametric test (Student's t test).

The confidence interval throughout the study was 95% (and α=0.05).

The comparison between the computer system and the trained observer was coded as True Positive (TP), False Positive (FP), True Negative (TN), and False Negative (FN).

The kappa (K) value was applied to measure the agreement between the computer system and the trained observer.

### Defining the groups - primary snorers and snorers with OSAHS

After trained observers using the JBioSignal Viewer validated all exams, we separated each event that was classified as a snore into two groups. One group consisted of primary snorers in which the apnea/hypopnea index (AHI) was below 5; the other group consisted of snorers with OSAHS (AHI ≥ 5).

It should be noted that the total number of snoring events was the sum of events classified by the algorithm (TP) added to the snores that were classified as such by trained observers (FN). The duration of each snoring event was shown in the JBioSignal Viewer.

## RESULTS

### Description of the study population

Table 1 shows the descriptive statistics of each variable in this study.

**Table 1:** Descriptive statistics of the study population.

	Summary of the Study Variables				
Variables	Minimum	Median	Maximum	Mean	Standard Deviation
Demography					
Age (years)	5	41	64	40,9	12,13
Weight (kg)	19	96	135	92,06	26,63
Height (m)	1,09	1,77	1,95	1,73	0,17
BMI (kg/m2)	15,99	29,3	50,19	30,27	6,98
Gender					
Male	23 (74%)				
Female	8 (26%)				
Total no. of patients (n)	31 (100%)				
Information about Polysomnography					
Sleep efficiency	24	81	95	75,77	16
AHI	0	7,62	127,9	19,33	29,7
No. obstructive apneas	0	7	546	36,06	120,5
Mean saturation (%)	87	93	96	93,13	2,22
No. awakenings	3	10	59	14,16	11,07
No. awakenings > 5 min	2	8	17	8,16	3,82
Subjective perception - snorer					
Yes	27 (87%)				
No	4 (13%)				
Diagnosis of obstructive apnea (AHI)					
Normal [0 - 5)	13 (42%)				
Mild [5 - 16)	6 (19%)				
Moderate[16 - 31)	8 (26%)				
Intense (>= 31)	4 (13%)				

### Validating the automatic snore detector

Table 2 presents a comparison of classifications made by trained observers and the computer algorithm. There were 43,976 snores; the sensitivity of the computer program was 99.26% (ranging from 89.74% to 100%), the specificity was 97.35% (ranging from 59.57% to 99.74%), the accuracy was 98.24%, the positive predictive value was 97%, and the negative predictive value was 99.34%. The agreement between the computer system and trained observers was 0.96 (kappa).

**Table 2:** Evaluation of the snore detector.

Validation of the Snore Detector									
Exam	SJT (snorer?)	No. of snores	TP	FP	TN	FN	Sensitivity	Specificity	Accuracy
EX01	No	22	21	76	112	1	95.45	59.57	63.33
EX02	No	866	865	25	965	1	99.88	97.47	98.60
EX03	Yes	656	656	15	759	0	100.00	98.06	98.95
EX04	Yes	2797	2789	52	3081	8	99.71	98.34	98.99
EX05	Yes	293	286	22	345	7	97.61	94.01	95.61
EX06	Yes	1893	1885	211	2252	8	99.58	91.43	94.97
EX07	No	90	90	25	149	0	100.00	85.63	90.53
EX08	Yes	234	210	20	314	24	89.74	94.01	92.25
EX09	Yes	826	819	54	934	7	99.15	94.53	96.64
EX10	No	32	29	164	242	3	90.63	59.61	61.87
EX11	Yes	932	924	28	1074	8	99.14	97.46	98.23
EX12	Yes	880	860	22	998	20	97.73	97.84	97.79
EX13	Yes	293	286	22	345	7	97.61	94.01	95.61
EX14	Yes	1140	1124	46	1308	16	98.60	96.60	97.51
EX15	Yes	1649	1639	14	1775	10	99.39	99.22	99.30
EX16	Yes	2136	2127	67	2441	9	99.58	97.33	98.36
EX17	Yes	1240	1237	17	1301	3	99.76	98.71	99.22
EX18	No	21	21	24	55	0	100.00	69.62	76.00
EX19	Yes	4213	4209	28	4483	4	99.91	99.38	99.63
EX20	Yes	1467	1457	16	1597	10	99.32	99.01	99.16
EX21	Yes	4129	4065	28	4493	64	98.45	99.38	98.94
EX22	Yes	2617	2601	14	2905	16	99.39	99.52	99.46
EX23	Yes	354	353	17	437	1	99.72	96.26	97.77
EX24	Yes	729	723	25	800	6	99.18	96.97	98.01
EX25	Yes	4619	4589	61	4908	30	99.35	98.77	99.05
EX26	Yes	1624	1622	28	1789	2	99.88	98.46	99.13
EX27	Yes	1090	1075	19	1239	15	98.62	98.49	98.55
EX28	Yes	3911	3903	11	4144	8	99.80	99.74	99.76
EX29	Yes	1530	1525	68	1676	5	99.67	96.10	97.77
EX30	Yes	1315	1300	12	1497	15	98.86	99.20	99.04
EX31	Yes	378	360	100	560	18	95.24	84.85	88.63
Total		43976	43650	1331	48978	326	99.26	97.35	98.24

SJT - Subjective judgment of the polysomnography technician during the exam (snorer?); TP - true positive; TN - true negative; FP - false positive; FN - false negative

### Duration of snoring episodes in obstructive sleep apnea patients

Table 3 shows a descriptive analysis of the duration of snoring episodes (in seconds), separated into two groups: a group of obstructive sleep apnea syndrome patients and a group of primary snorers.

**Table 3:** Analysis of the duration, in seconds, of snoring episodes.

Descriptive analysis of the duration of snoring		
	OSAHS patients	Primary snorers
No. of snores (n)	9993	7691
Minimum duration	0.55	0.55
Median	0.8	0.9
Maximum duration	2.95	2.55
Mean	0.8636	1.002
Standard deviation	0.271	0.3562
Standard error	0.002711	0.004062
Confidence interval (95%)	[0.8583 0.8689]	[0.9944 1.01]

OSAHS with AHI >=5 (information extracted from the official report) Primary snorers with AHI < 5.

The t test was applied in both groups to check for mean differences in the duration of snoring episodes. The result was a statistically significant difference (*p*<0.0001).

## DISCUSSION

This study presents a description and evaluation of an algorithm to detect snoring episodes during sleep by applying a time analysis of electric signals that are equivalent to snoring sound signals.

In recent years, several researchers have analyzed snoring sounds under the same light as voice recognition because of their physiologic similarities and available digital processing and analytical methods. The analogy between snoring and voice stems from their common origin in the vocal tract^20^. However, voice recognition techniques have not been widely adopted in clinical practice. Pevernagie^4^ argues that the acoustic analysis of snoring is still in its initial phases as medical science, and few techniques are applicable in the clinical setting.

Karunajeewa^13^ applied linear predictive coding (LPC), zero passage rates, signal energy, and normalized correlation coefficient for segmenting and classifying sound signals to detect snoring and neglect silence and other types of signals. This computer algorithm attained 90.74% accuracy.

Cavusoglu's^11^ algorithm applied 500 Hz sub-band energy distribution in segments (or samples) of the snoring sound signal and attained 97.3% accuracy in primary snorers and 86.8% in OSAHS patients. Duckitt^12^ applied Hidden Markov Models (HMM) and attained 89% accuracy.

These abovementioned techniques require a high sampling rate (>7500 Hz) to process snoring sounds. The algorithm presented here consists of a time analysis (T_duration_) used the snoring electric signal (in 31 patients) at a low sampling rate (200 Hz) to attain 96% accuracy compared to the gold standard (trained observer). This is evidence that electric signal processing - even at a low sampling rate - may be used in detecting snores automatically.

An issue when validating snores manually is that its definition varies among observers. Hoffstein^17^ showed that it was difficult to objectively define and quantify snores because of the subjective perception of listeners. These authors reviewed 25 polysomnographies (all lasting the entire night and recording snores). Snoring sounds were simultaneously recorded on a cassette tape and paper. An experienced polysomnography technician objectively counted the number of snores during 20 minutes of examination time. Two other polysomnography technicians listened to the snores and counted their perception of snores. The results revealed that in 7 of 25 patients, the difference between the first technician (objective counting of snores) and the other two technicians (subjective counting of snores) was over 25%. In another group, the difference among technicians in 7 of 25 patients was over 25%. The kappa agreement among listeners was 0.49 (moderate). Hoffstein concluded that judging snoring events is highly subjective.

In our study, the agreement between trained observers and the computer system was 0.96 (kappa), a significant agreement. This finding suggests that automatic snore detection techniques may be adopted in clinical practice, even if the sampling rate of the electric signal of snoring is low.

A 0.96 kappa value is evidence that a computer system may perform the role of human observers (the polysomnography technician) for classifying snoring episodes. Agreement among humans - which is the gold standard - reaches a kappa agreement level of only 0.49 (moderate).

We found that the mean duration of snoring events in OSAHS patients was shorter compared to plain snorers, as shown in Student's t test (*p*<0.0001). This finding suggests that shorter snoring events in OSAHS patients should be carefully investigated.

## CONCLUSION

The algorithm presented above may be applied in the following clinical tasks:

1) Automatically identifying, using a low sampling rate, electric signals that are equivalent to the snoring sound signals.
2) Monitoring the progression of therapy for respiratory sleep disorders, in particular snoring, using statistical comparisons before and after treatment.

As is often the case in polysomnography clinics, the reports describe only subjective impressions made by technicians during the exam (intense snoring, moderate snoring, and mild snoring). This algorithm facilitates generating quantitative reports based on electric recordings of snoring; it is reproducible, reliable, potentially useful, and reduces manual labor and costs.
